# Identification and Severity Determination of Wheat Stripe Rust and Wheat Leaf Rust Based on Hyperspectral Data Acquired Using a Black-Paper-Based Measuring Method

**DOI:** 10.1371/journal.pone.0154648

**Published:** 2016-04-29

**Authors:** Hui Wang, Feng Qin, Liu Ruan, Rui Wang, Qi Liu, Zhanhong Ma, Xiaolong Li, Pei Cheng, Haiguang Wang

**Affiliations:** 1 Department of Plant Pathology, China Agricultural University, Beijing, China; 2 Kaifeng Experimental Station of China Agricultural University, Kaifeng, Henan Province, China; College of Agricultural Sciences, UNITED STATES

## Abstract

It is important to implement detection and assessment of plant diseases based on remotely sensed data for disease monitoring and control. Hyperspectral data of healthy leaves, leaves in incubation period and leaves in diseased period of wheat stripe rust and wheat leaf rust were collected under in-field conditions using a black-paper-based measuring method developed in this study. After data preprocessing, the models to identify the diseases were built using distinguished partial least squares (DPLS) and support vector machine (SVM), and the disease severity inversion models of stripe rust and the disease severity inversion models of leaf rust were built using quantitative partial least squares (QPLS) and support vector regression (SVR). All the models were validated by using leave-one-out cross validation and external validation. The diseases could be discriminated using both distinguished partial least squares and support vector machine with the accuracies of more than 99%. For each wheat rust, disease severity levels were accurately retrieved using both the optimal QPLS models and the optimal SVR models with the coefficients of determination (*R*^2^) of more than 0.90 and the root mean square errors (RMSE) of less than 0.15. The results demonstrated that identification and severity evaluation of stripe rust and leaf rust at the leaf level could be implemented based on the hyperspectral data acquired using the developed method. A scientific basis was provided for implementing disease monitoring by using aerial and space remote sensing technologies.

## Introduction

Stripe rust caused by *Puccinia striiformis* f. sp. *tritici* (*Pst*) and leaf rust caused by *P*. *recondita* f. sp. *tritici* (*Prt*) are two kinds of economically important airborne diseases of wheat around the world [1–3]. In China, severe yield losses of wheat can be induced by these two diseases with large occurrence areas and high epidemic frequency. Therefore, they both are important factors affecting the food security production in China [1, 4–6]. Both wheat stripe rust and wheat leaf rust can cause appearance of uredinia on wheat leaves, and the symptoms caused by these two diseases are similar. Urediospores produced by the uredinia can infect wheat leaves [1, 6]. Relying on urediospores, wheat stripe rust and wheat leaf rust can accomplish multiple disease cycles in the field [1, 6]. Thus, it is of great significance to make accurate identification and timely monitoring of these two diseases for prediction and control of the diseases. At the present time, diagnosis, identification and monitoring of the two diseases are mainly accomplished by plant protection technical personnel through the in-field observation and survey according to the visible disease symptoms. It is a labor-intensive and time-consuming process with low efficiency, and sometimes it is easy to make some mistakes. Thus, timely control and management of the diseases would be affected. Studies on application of molecular biological techniques to diagnose and identify the diseases have been reported [7–10]. However, it is difficult to promote the techniques to the practical application because of high technical requirements and great operation difficulty. So it is significant to explore a method for rapid and accurate diagnosis, identification and quantitative assessment of the diseases.

With the rapid development of remote sensing technologies, related technologies have been increasingly applied in the agricultural field. Studies on monitoring plant diseases based on remote sensing technologies have also been increasing. As a rapid, accurate, nondestructive and economical technology, the hyperspectral remote sensing technology could provide strong technical supports for the monitoring of plant diseases [11–13]. So far, mostly studies focused on detecting and monitoring of single plant disease using remote sensing techniques, and only a few studies based on remote sensing technologies have been reported for the identification and monitoring of two or more than two kinds of plant diseases or plant pests. Remote sensing techniques are mostly utilized in the studies on detecting and monitoring of plant diseases at the leaf and canopy scales, and there are only a few studies on the identification and monitoring of plant diseases based on aerial remote sensing technologies or space remote sensing technologies [12–15].

Studies on detection, identification and monitoring of wheat stripe rust using remote sensing technologies at the leaf, canopy and region scales have been conducted [15]. A study conducted by Huang et al. [16] showed that the spectral reflectance of individual wheat leaves infected with *Pst* was basically positively correlated with disease severity in the band of 376–1600 nm. Especially, the correlation reached a significant level in the bands of 446–725 nm and 1380–1600 nm, thus these two bands were selected as the sensitive bands to the severity of wheat stripe rust. And the regression models built in the study could accurately determine the disease severity. A study on spectral characteristics of single wheat leaf infected with *Pst* conducted by An et al. [17] showed that the spectral reflectance increased with the increase of disease severity and that the correlation between spectral reflectance and severity of wheat stripe rust was the highest in the band of 670–690 nm. Moreover, a regression model for severity inversion of wheat stripe rust was built with spectral reflectance as the independent variable. Based on the hyperspectral data of healthy wheat leaves and *Pst*-infected wheat leaves in a range of disease severity levels, Wang et al. [18] applied support vector machine (SVM) to classify and identify the severity of wheat leaves, and high identification accuracy was obtained. Zhao et al. [19] built two linear regression models with photochemical reflectance index (PRI) for severity inversion of wheat stripe rust at the leaf scale. Based on the in-field spectral reflectance of wheat at the canopy level, Moshou et al. [20] analyzed the differences of spectral reflectance between healthy wheat plants and diseased wheat plants infected with *Pst* in the early stage in the development of wheat stripe rust. In the study, a discrimination model built by quadratic discriminant analysis could detect the disease with a performance of approximately 95%, and better results with a performance of better than 99% were obtained by using a neural network. Canopy spectra of wheat infected with *Pst* at different growth stages were acquired and the synchronous investigation of disease index was performed by Huang et al. [21]. The results showed that the disease index was correlated well with the canopy reflectance in the bands of 630–687 nm, 740–890 nm and 976–1350 nm that were regarded as the sensitive bands for monitoring wheat stripe rust. It was also noted that the disease index of wheat stripe rust was significantly correlated with the canopy reflectance at 930 nm with a correlation coefficient higher than 0.90 [22]. From the unmanned aerial vehicle images of wheat stripe rust with different disease indices, Leng et al. [23] extracted the reflectance in the red band, the green band and the blue band and the canopy reflectance, and demonstrated significant correlation between disease index and each kind of the reflectance. The results of the study showed that disease index of wheat stripe rust could be well fitted by using the model built with each kind of the reflectance. Based on multi-temporal airborne hyperspectral images of a wheat field acquired by using a pushbroom hyperspectral imager designed by the Chinese Academy of Science (CAS) on a Yun-5 aircraft, disease prevalence and diseased regions of wheat stripe rust were successfully remotely sensed [24–27]. Great efforts were made to investigate the potential of space remote sensing for monitoring wheat stripe rust [28–32]. However, it is still a long way to implement detection and monitoring of wheat stripe rust by using satellite images, and more efforts should be made.

However, only several studies on remote sensing monitoring of wheat leaf rust have been reported. Based on hyperspectral data in the range of 450–1000 nm at the leaf scale, Ashourloo et al. [33] evaluated the effects of different symptoms of wheat leaf rust on 22 spectral vegetation indices (SVIs). The results of the study showed that the scattering of all SVI values increased with the increase of disease severity and that few indices had the ability to indirectly detect the disease. Ashourloo et al. [34] developed two spectral disease indices on the reflectance of wheat leaves at the 605, 695 and 455 nm wavelengths for detection of wheat leaf rust with high accuracy.

Wheat stripe rust and wheat leaf rust often occur simultaneously or simultaneously with other diseases and pests in the field. Therefore, it is necessary to conduct the studies on discriminating wheat stripe rust and wheat leaf rust and on discriminating these two diseases from other wheat diseases and pests based on remote sensing technologies. To the best of our knowledge, at this present time, there are few reports on the detection and assessment of two or more than two kinds of wheat diseases and pests based on remote sensing technologies. Based on the reflectance spectra acquired from individual wheat leaves, Devadas et al. [35] evaluated the ability of ten widely-used vegetation indices to discriminate the leaves infected with stripe rust, leaf rust and stem rust caused by the pathogen *Puccinia graminis* f. sp. *tritici*. It was reported that no single index could discriminate the three kinds of wheat rusts from each other. It was suggested that the anthocyanin reflectance index could be used to discriminate healthy leaves, leaves infected with stripe rust and mixed leaves infected with leaf rust and stem rust, and the transformed chlorophyll absorption and reflectance index could subsequently be used to separate the leaves infected with leaf rust and those infected with stem rust. Based on the spectra of individual wheat leaves infected with stripe rust and powdery mildew (caused by *Blumeria graminis* f. sp. *tritici*), Yuan et al. [36] discriminated the two diseases with the overall accuracy more than 80% by using Fisher linear discriminant analysis (FLDA) and retrieved disease severity for each disease with the root mean square error (RMSE) less than 15% by using partial least square regression (PLSR). At the leaf scale, Huang et al. [37] designed four new spectral indices to identify healthy wheat leaves and wheat leaves infested with powdery mildew, stripe rust and aphids with classification accuracies of 86.5%, 85.2%, 91.6%, and 93.5%, respectively. A study was also conducted by Yuan et al. [38] for discriminating healthy wheat leaves and wheat leaves infested with powdery mildew, stripe rust and aphids and for estimating stress intensity based on the reflectance spectra at the leaf scale. The satisfactory discrimination performance was obtained with an overall accuracy of 0.75 using the discrimination model built by FLDA and the reasonable estimates were achieved with a coefficient of determination (*R*^2^) of 0.73 and a RMSE of 0.148 using the estimating model built by PLSR. Based on the hyperspectral data at the canopy level, Qiao et al. [39] identified the damage symptoms caused by powdery mildew, stripe rust and wheat aphids on winter wheat with the accuracies more than 90% by using stepwise discriminate analysis and hierarchical clustering, respectively. Wang et al. [40] implemented identification and disease index inversion of wheat stripe rust and wheat leaf rust based on hyperspectral data at the canopy scale with satisfactory accuracies. Franke and Menz [41] performed the in-field detection of powdery mildew and leaf rust in wheat based on three high-resolution remote sensing images and the overall classification accuracies of 56.8%, 65.9% and 88.6% were obtained for each image, respectively. It was indicated that the high-resolution remote sensing images were moderately suitable for early detection of different diseases.

Both remote sensing monitoring of plant diseases at the canopy level and monitoring plant diseases by aerial and space remote sensing are influenced by environmental factors such as the geometric structure of the vegetation canopy, soil background and atmosphere [13, 15]. In contrast, spectral measurements at the leaf level are more accurate and more reliable. However, spectral measurements at the leaf level are usually performed under indoor illumination conditions which have great differences with nature light source. Moreover, the measurements have high requirements for experimental equipments and usually require an additional device such as integrating sphere or leaf clip which has its own light source. Spectral data in the reported studies on remote sensing monitoring of wheat stripe rust at the leaf level were basically acquired based on the use of an integrating sphere [16–18] or a leaf clip [19, 36, 38]. It is very difficult to accurately measure spectral data at the leaf level under outdoor conditions. Usually, plant leaves are detached and preserved in some way, and then are brought back for the indoor measurements. Some changes in water content and cell structure of the leaves are inevitably induced in this process, and these changes would affect the accuracy of the acquired spectral data. It is very important to explore a simple, low-cost and accurate method suitable for in-field acquisition of spectral data at the leaf level.

In this study, using a proposed hyperspectral data measuring method named as black-paper-based measuring method, the hyperspectral data of healthy wheat leaves, wheat leaves in incubation period and wheat leaves in diseased period of stripe rust and leaf rust were collected under natural environmental conditions in the field. The models to identify wheat stripe rust and leaf rust were built using distinguished partial least squares (DPLS) and SVM, respectively. And the disease severity inversion models of each individual disease were built using quantitative partial least squares (QPLS) and support vector regression (SVR), respectively. Feasibility of the identification and assessment of wheat stripe rust and leaf rust based on the acquired hyperspectral data at the leaf level by using the proposed method was investigated. The aims of this study were to provide a simple and convenient method for obtaining hyperspectral data at the leaf level and to provide a method for the monitoring and assessment of the two wheat diseases.

## Materials and Methods

### Wheat Cultivation and Artificial Spray Inoculation

Wheat variety Beijing 0045, moderately susceptible to *Pst* and *Prt*, was selected as the experimental cultivar. In the fall of 2013, the seeds of the cultivar were sown in the experimental field located in Kaifeng Experimental Station of China Agricultural University, Kaifeng, Henan Province, China. Two large zones (Zone 1 and Zone 2) were divided from the experimental field. Zone 1 was used as the experimental zone of wheat stripe rust and Zone 2 was used as the experimental zone of wheat leaf rust. Each zone was divided into 18 plots and the size of each plot was 3 m × 4 m. Between the plots, wheat variety Nongda 195, which is highly resistant to *Pst* and has slow rusting resistance to *Prt*, was planted as protective belts.

Using the method of Cheng et al. [42], the urediospores of *Pst* and *Prt* for artificial spray inoculation in the experimental field were multiplied on wheat cultivar Mingxian 169 which is susceptible to both *Pst* and *Prt* in an artificial climate chamber in the Lab of Plant Disease Epidemiology, Department of Plant Pathology, China Agricultural University. For multiplication of the two kinds of wheat rust pathogens, artificial spray inoculation was conducted when the first leaves of wheat seedlings fully expanded in the artificial climate chamber at 11–13°C and 60–70% relative humidity (RH) with 12 h light per day (10000 lux). Urediospores of *Pst* or *Prt* stored in a liquid nitrogen container were taken out, reactivated in warm water of 40°C for 5 min, and then hydrated at 4°C for 12 h. A spore suspension was made with 0.2% Tween 80 solution. After wax on leaf surface was removed by rubbing the surface using fingers dipped with sterile water, about 20 seedlings of each pot (10 cm in diameter) were uniformly sprayed with the spore suspension. The inoculated seedlings were immediately placed into a moist chamber in dark conditions at 11–13°C for 24 h, and then transferred into the artificial climate chamber under the conditions described above. After 15 days, urediospores were collected by shaking them into a finger-type tube. Some collected urediospores were used to inoculate the new wheat seedlings for multiplication of *Pst* or *Prt*. The rest were packed into the cryogenic vials and then preserved in the liquid nitrogen container for the following experiments.

In the experimental field, the artificial spray inoculation of *Pst* and *Prt* was performed late in the afternoon in April 2014. To achieve different disease prevalence of wheat stripe rust and wheat leaf rust in the plots, the *Pst* spore suspensions of 100 mg/L, 80 mg/L, 60 mg/L, 40 mg/L and 20 mg/L were made with 0.2% Tween 80 solution, and the *Prt* spore suspensions of 50 mg/L, 40 mg/L, 30 mg/L, 20 mg/L and 10 mg/L were made with 0.2% Tween 80 solution. The control plots were not inoculated using the pathogens. In each zone, each treatment was replicated three times and the experiment was performed with randomized block design. Each plot needed to be inoculated was evenly sprayed with the corresponding spore suspensions using a sprayer. Just before inoculation, a sprayer was used to make the wheat leaves covered with water droplets and the wax on the leaf surface was removed by rubbing the surface using fingers. For moisturizing the wheat leaves, the inoculated plot was immediately covered with a plastic film that was sprayed with water, and then the edges of the plastic film were covered with earth. The plastic films were removed between 8: 00 and 9: 00 (Beijing Time) in the next day. The disease symptoms appeared on 20 days after inoculation.

### Acquisition of Hyperspectral Data

A novel hyperspectral data measuring method named as black-paper-based measuring method, was developed in this study. Firstly, under in-field natural conditions, the collected fresh wheat leaves were fixed on a piece of black paper (30 cm × 20 cm) with transparent double-sided adhesive tape. The leaves were arranged side by side, and the number of leaves for each group could be determined according to the measuring area. In this study, five wheat leaves in the same level of disease severity was treated as a group, and the total leaf width of each group was about 10 cm. Six groups of wheat leaves were pasted on a piece of black paper. When the leaves were fixed on the paper, the upper (adaxial) surfaces of the leaves were on the upside because the uredinia were on the upper surfaces of diseased leaves. The hyperspectral data of wheat leaves in the wavelength range of 325–1075 nm were measured by using an ASD spectrometer (ASD FieldSpec HandHeld 2) (ASD Inc., Boulder, Colorado, USA) with a 25° field-of-view, a wavelength accuracy of ±1 nm, a spectral resolution of <3 nm at 700 nm and minimum integration time of 8.5 ms. The acquired spectral data were processed by using the softwares ViewSpecPro (Version 6.0.11) and MATLAB (R2013b). All hyperspectral measurements were performed on clear and sunny days between 10: 00 and 14: 00 (Beijing Time). When the hyperspectral measurement was made, the sensor of the spectrometer was vertically positioned at a height of 8 cm above the fixed leaves. Three spectra were measured for the leaves of each group, and the average value was treated as the spectrum of the group at the leaf level. White board correction was made after hyperspectral measurements of three groups of wheat leaves.

Before artificial spray inoculation of *Pst* and *Prt* in the experimental field, the spectra of 180 groups of healthy wheat leaves were measured, and 180 spectra were obtained for further analysis and modeling after averaging the three spectra of each group. On the tenth day after inoculation, no visible disease symptoms appeared on the wheat leaves inoculated with the pathogens, and the leaves were in the incubation period of wheat stripe rust or wheat leaf rust. On that day, for each kind of wheat rust, the hyperspectral measurements of 180 groups of wheat leaves in the incubation period were made, and 540 spectra were obtained. After averaging the three spectra of each group, 180 spectra in the incubation period of each kind of wheat rust were used for analysis and modeling. After disease symptoms appeared on the wheat leaves, the diseased wheat leaves were collected and the severity levels of wheat stripe rust and leaf rust were assessed according to the Rules for Monitoring and Forecast of the Wheat Stripe Rust (*Puccinia striiformis* West.) (National Standard of the People’s Republic China, GB/T 15795–2011) and the Rules for the Investigation and Forecast of Wheat Leaf Rust (*Puccinia recondita* Rob. *et* Desm.) (Agricultural Industry Standard of the People’s Republic of China, NY/T 617–2002), respectively. The disease severity of the diseased leaf infected with *Pst* or *Prt* was estimated as 1%, 5%, 10%, 20%, 40%, 60%, 80% or 100%. For each kind of wheat rust, 15 groups of wheat leaves at each severity level were collected and then 120 groups were totally obtained. Subsequently, 360 spectra of the diseased leaves for each disease were acquired, and after averaging the three spectra of each group, 120 spectra of the leaves infected with *Pst* or *Prt* were obtained for the corresponding analysis.

### Preprocessing Hyperspectral Data

In this study, modeling was conducted using the spectral reflectance data in the three regions including the acquired full spectral region (325–1075 nm) (named as original spectral reflectance data), the visible region (380–780 nm) and the near infrared region (780–1075 nm). It was reported that the influence of background noises and non-ideal information could be removed from the original spectra by derivative spectrometry and that first derivative spectra, second derivative spectra and high order derivative spectra could work well for the reduction of low-frequency background noises and the resolution of overlapping spectra [43]. It was also demonstrated that logarithmic transformation of the reciprocals of the spectral reflectance could increase the differences among the spectra in the visible region and reduce the influence of environmental factors such as the changes of light conditions on acquired spectra [44]. Therefore, to reduce the effects of noise signals on spectral information, first derivatives of the original spectral reflectance, second derivatives of the original spectral reflectance and the logarithms of the reciprocals of the original spectral reflectance were calculated in this study and then applied for modeling. In addition, a spectral feature set consisting of 22 spectral feature parameters [11] (as shown in Table 1) was built to select spectral feature parameters for modeling. To reduce the number of spectral feature parameters for modeling, correlation analysis between each spectral feature parameter and disease severity of the corresponding disease was performed, and the spectral feature parameters were selected for modeling as the corresponding correlation coefficients (*r*) for the two diseases were both higher than 0.5, 0.6, 0.7 or 0.8. The selected results of the spectral feature parameters were shown in Table 2. Identification models and severity inversion models of wheat stripe rust and wheat leaf rust based on the original spectral reflectance data, the original spectral reflectance in the visible region, the original spectral reflectance in the near infrared region, the first derivatives of the original spectral reflectance, the second derivatives of the original spectral reflectance, the logarithms of the reciprocals of the original spectral reflectance and the selected spectral feature parameters, respectively, and the effects of the different preprocessing methods of spectral data on modeling were compared.

**Table 1:** The spectral feature parameters used in this study.

Feature parameter	Definition or calculation formula
*D*b	The maximum of first derivatives of spectral reflectances within blue edge (490–530 nm)
*λ*b	The wavelength at the position of *D*b (nm)
*D*y	The maximum of first derivatives of spectral reflectances within yellow edge (550–582 nm)
*λ*y	The wavelength at the position of *D*y (nm)
*D*r	The maximum of first derivatives of spectral reflectances within red edge (680–780 nm)
*λ*r	The wavelength at the position of *D*r (nm)
*R*g	The maximum reflectance in 510–560 nm
*λ*g	The wavelength at the position of *R*g (nm)
*R*r	The minimum reflectance in 640–680 nm
*λ*_0_	The wavelength at the position of *R*r (nm)
*SD*b	The sum of first derivatives of spectral reflectances within blue edge (490–530 nm)
*SD*y	The sum of first derivatives of spectral reflectances within yellow edge (550–582 nm)
*SD*r	The sum of first derivatives of spectral reflectances within red edge (680–780 nm)
*R*g/*R*r	The ratio of *R*g to *R*r
(*R*g-*R*r)/(*R*g+*R*r)	The normalized value of *R*g and *R*r
*SD*r/*SD*b	The ratio of *SD*r to *SD*b
*SD*r/*SD*y	The ratio of *SD*r to *SD*y
(*SD*r-*SD*b)/(*SD*r+*SD*b)	The normalized value of *SD*r and *SD*b
(*SD*r-*SD*y)/(*SD*r+*SD*y)	The normalized value of *SD*r and *SD*y
Normalized difference vegetation index (NDVI)	$\frac{{\text{R}}_{\text{Nir}}-{\text{R}}_{\text{Red}}}{{\text{R}}_{\text{Nir}}+{\text{R}}_{\text{Red}}}$
Ratio vegetation index (RVI)	$\frac{{\text{R}}_{\text{Nir}}}{{\text{R}}_{\text{Red}}}$
Difference vegetation index (DVI)	*R*_*Nir*_−*R*_*Red*_

**Table 2:** The selected hyperspectral parameters by using different criteria based on the correlation coefficients (*r*).

Selection criteria	The selected spectral feature parameters
*r*>0.5	*R*g, *R*r, *R*g/*R*r, (*R*g-*R*r) / (*R*g+*R*r), NDVI and RVI
*r*>0.6	*R*g, *R*r, NDVI and RVI
*r*>0.7	*R*r, NDVI and RVI
*r*>0.8	NDVI

### Establishment of Identification Models of Wheat Stripe Rust and Leaf Rust

The models to discriminate five categories of wheat leaves including healthy leaves, leaves in the incubation period of stripe rust, leaves in the diseased period of stripe rust, leaves in the incubation period of leaf rust and leaves in the diseased period of leaf rust, were constructed by using two methods including DPLS and SVM, respectively. The models were validated by using two methods including leave-one-out cross validation (LOOCV) and external validation. Each model was evaluated by using the identification accuracy. When modeling was performed based on external validation, the acquired spectral data were randomly divided into training set and testing set as the ratio of training set to testing set was equal to 1:1, 2:1, 3:1, 4:1 or 5:1, and the effects of different ratios on modeling were compared.

### Establishment of Severity Inversion Models of Wheat Stripe Rust and Leaf Rust

Severity inversion models of wheat stripe rust were established by using two methods including QPLS and SVR, respectively. Severity inversion models of wheat leaf rust were also established by using QPLS and SVR, respectively. All the severity inversion models were validated by LOOCV and external validation. The values of *R*^2^ and RMSE were used to evaluate the models. When modeling was performed based on external validation, the acquired spectral data were divided into training set and testing set with the ratio of training set to testing set equal to 1:1, 2:1, 3:1, 4:1 or 5:1 by using the content-grads method [45], and the effects of different ratios on modeling were compared.

## Results

### Spectral Characteristics of Stripe Rust and Leaf Rust in Wheat

**Fig 1:**
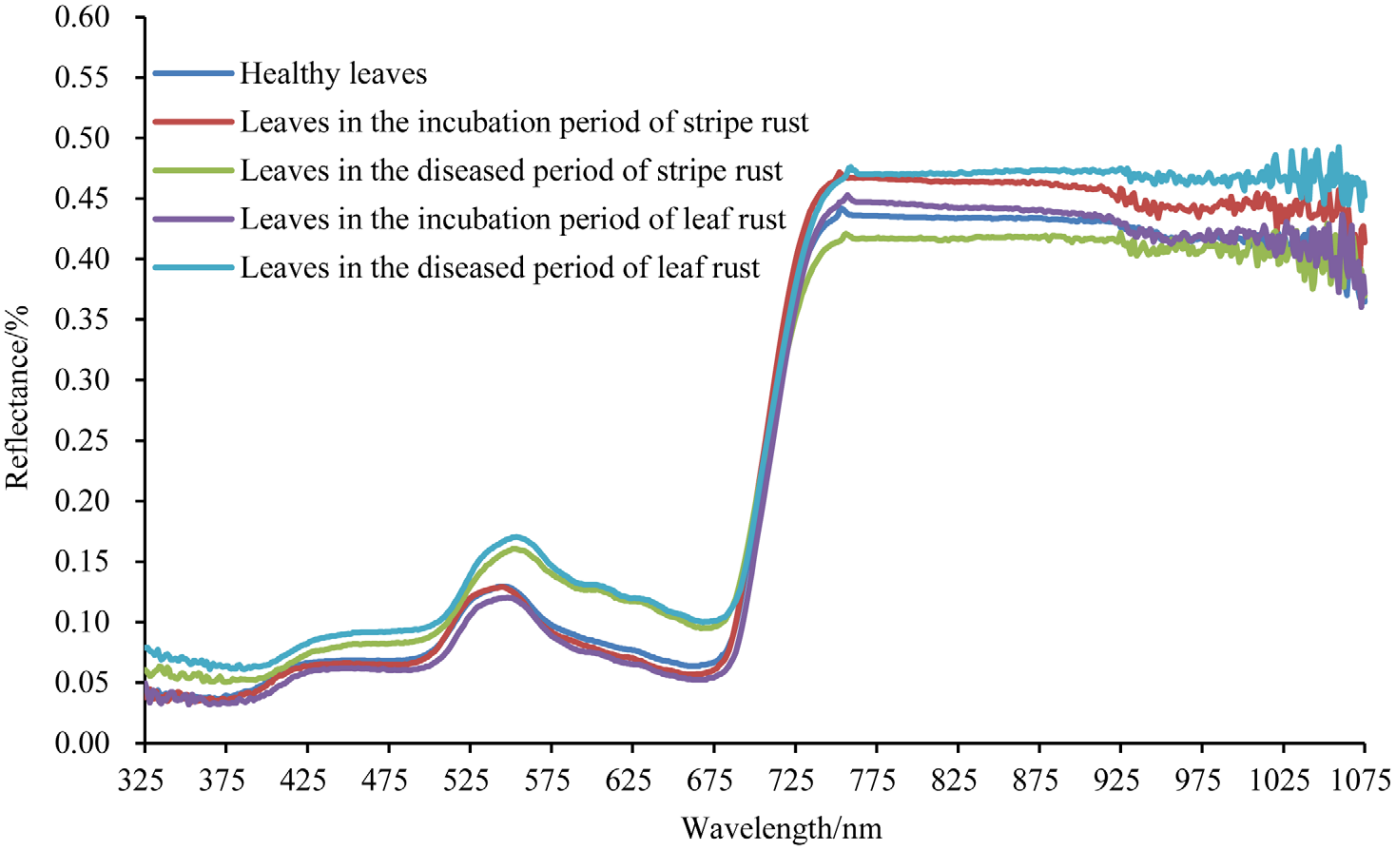
The spectra of different classes of wheat leaves.

After averaging the spectral data of healthy wheat leaves, wheat leaves in the incubation period of stripe rust, wheat leaves in the diseased period of stripe rust, wheat leaves in the incubation period of leaf rust and wheat leaves in the diseased period of leaf rust according to their categories, respectively, the spectrum of each category was obtained and the corresponding spectral curve was showed in Fig 1. As shown in Fig 1, for both wheat stripe rust and leaf rust, compared with the spectral reflectance of the healthy wheat leaves in the range of 325–700 nm, the spectral reflectance of wheat leaves in diseased period increased to some extent, and the spectral reflectance of wheat leaves in the incubation period relatively decreased. Moreover, in the acquired full spectral region of 325–1075 nm, the reflectance values of wheat leaves in the incubation period of stripe rust were higher than that of wheat leaves in the incubation period of leaf rust, and the reflectance values of wheat leaves in the diseased period of stripe rust were lower than that of wheat leaves in the diseased period of leaf rust. The differences in the near infrared region were more obvious than in the visible region.

### Identification of Wheat Stripe Rust and Leaf Rust Based on the Hyperspectral Data at the Leaf Level

#### Identification of Wheat Stripe Rust and Leaf Rust by Modeling Based on LOOCV

When LOOCV was used as the validation method, the identification accuracies of the identification models of wheat stripe rust and leaf rust built based on the original spectral reflectance data, the original spectral reflectance in the visible region, the original spectral reflectance in the near infrared region, the first derivatives of the original spectral reflectance, the second derivatives of the original spectral reflectance, the logarithms of the reciprocals of the original spectral reflectance and the selected spectral feature parameters, respectively, were shown in Table 3. As shown in Table 3, the identification accuracies of more than 94% were obtained for the disease identification DPLS models and the disease identification SVM models built based on the original spectral reflectance data, the original spectral reflectance in the visible region, the original spectral reflectance in the near infrared region, the first derivatives of the original spectral reflectance, the second derivatives of the original spectral reflectance and the logarithms of the reciprocals of the original spectral reflectance, respectively. Among the disease identification DPLS models, the model built based on the logarithms of the reciprocals of the original spectral reflectance achieved the highest identification accuracy of 99.87%. Among the disease identification SVM models, the model built based on the first derivatives of the original spectral reflectance, the second derivatives of the original spectral reflectance or the logarithms of the reciprocals of the original spectral reflectance achieved the highest identification accuracy of 99.23%. The identification accuracies of the disease identification DPLS models and the disease identification SVM models based on the selected spectral feature parameters, respectively, were all very low. Moreover, with the increase of the selection criteria, the number of the selected spectral feature parameters was getting fewer, and the modeling effect was getting worse. Especially, as the correlation coefficients for individual wheat rusts were both higher than 0.8, only normalized difference vegetation index (NDVI) was selected for modeling. And for the disease identification DPLS model and the disease identification SVM model based on NDVI, the lowest identification accuracies of 38.08% and 47.31% were obtained, respectively.

**Table 3:** The results of identification models of wheat stripe rust and leaf rust by LOOCV.

Modeling data	Identification accuracy of DPLS model/%	Identification accuracy of SVM model/%
Original spectral reflectance data	99.74	99.21
Original spectral reflectance in the visible region	99.62	98.85
Original spectral reflectance in the near infrared region	98.33	94.49
First derivatives of the original spectral reflectance	99.62	99.23
Second derivatives of the original spectral reflectance	99.49	99.23
The logarithms of the reciprocals of the original spectral reflectance	99.87	99.23
Spectral feature parameters (*r*>0.5)	65.00	71.28
Spectral feature parameters (*r*>0.6)	64.36	71.03
Spectral feature parameters (*r*>0.7)	60.26	69.10
Spectral feature parameter (*r*>0.8)	38.08	47.31

#### Identification of Wheat Stripe Rust and Leaf Rust by Modeling Based on External Validation

When external validation was applied for modeling, the effects of different ratios between training sets and testing sets on the disease identification DPLS models and the disease identification SVM models were shown in Tables 4 and 5, respectively. As shown in Tables 4 and 5, the modeling ratios had slight effects on the disease identification models. The models built based on the original spectral reflectance data, the original spectral reflectance in the visible region, the original spectral reflectance in the near infrared region, the first derivatives of the original spectral reflectance, the second derivatives of the original spectral reflectance and the logarithms of the reciprocals of the original spectral reflectance, respectively, were better than that built based on the selected spectral feature parameters.

**Table 4:** The effects of different ratios between training sets and testing sets on the disease identification DPLS models.

Modeling data	The ratio of training set to testing set	Identification accuracy for training set/%	Identification accuracy for testing set/%
Original spectral reflectance data	1:1	99.74	99.74
	2:1	100.00	99.23
	3:1	99.83	99.49
	4:1	99.84	100.00
	5:1	99.85	100.00
Original spectral reflectance in the visible region	1:1	99.74	99.49
	2:1	99.81	99.62
	3:1	99.83	99.49
	4:1	99.84	100.00
	5:1	99.69	100.00
Original spectral reflectance in the near infrared region	1:1	99.74	95.90
	2:1	100.00	97.31
	3:1	99.83	96.92
	4:1	99.84	99.36
	5:1	99.69	97.69
First derivatives of the original spectral reflectance	1:1	100.00	98.72
	2:1	100.00	98.46
	3:1	100.00	98.97
	4:1	100.00	99.36
	5:1	100.00	98.46
Second derivatives of the original spectral reflectance	1:1	100.00	99.23
	2:1	100.00	98.46
	3:1	100.00	99.49
	4:1	100.00	98.72
	5:1	100.00	98.46
The logarithms of the reciprocals of the original spectral reflectance	1:1	100.00	99.49
	2:1	100.00	98.85
	3:1	100.00	99.49
	4:1	100.00	100.00
	5:1	100.00	100.00
Spectral feature parameters (*r*>0.5)	1:1	65.90	64.87
	2:1	65.77	65.77
	3:1	65.81	65.13
	4:1	65.22	66.03
	5:1	66.15	63.08
Spectral feature parameters (*r*>0.6)	1:1	64.36	64.62
	2:1	64.43	65.00
	3:1	64.79	65.13
	4:1	65.07	62.18
	5:1	64.92	62.31
Spectral feature parameters (*r*>0.7)	1:1	59.49	61.03
	2:1	60.58	60.00
	3:1	60.34	62.05
	4:1	60.42	62.18
	5:1	61.08	59.23
Spectral feature parameters (*r*>0.8)	1:1	38.72	38.72
	2:1	38.27	39.23
	3:1	40.34	37.95
	4:1	39.10	40.38
	5:1	39.23	38.46

**Table 5:** The effects of different ratios between training sets and testing sets on the disease identification SVM models.

Modeling data	The ratio of training set to testing set	Identification accuracy for training set/%	Identification accuracy for testing set/%
Original spectral reflectance data	1:1	99.74	97.69
	2:1	99.81	96.54
	3:1	99.83	98.97
	4:1	99.52	98.72
	5:1	99.85	99.23
Original spectral reflectance in the visible region	1:1	99.74	98.46
	2:1	100.00	98.85
	3:1	99.83	97.95
	4:1	99.84	100.00
	5:1	99.85	99.23
Original spectral reflectance in the near infrared region	1:1	99.74	93.33
	2:1	100.00	93.46
	3:1	99.83	93.33
	4:1	99.84	92.31
	5:1	99.85	96.15
First derivatives of the original spectral reflectance	1:1	100.00	97.95
	2:1	100.00	99.62
	3:1	100.00	99.49
	4:1	100.00	98.08
	5:1	100.00	100.00
Second derivatives of the original spectral reflectance	1:1	100.00	96.67
	2:1	100.00	95.77
	3:1	100.00	97.44
	4:1	100.00	96.15
	5:1	100.00	97.69
The logarithms of the reciprocals of the original spectral reflectance	1:1	100.00	99.74
	2:1	100.00	100.00
	3:1	100.00	99.49
	4:1	100.00	100.00
	5:1	100.00	100.00
Spectral feature parameters (*r*>0.5)	1:1	76.67	71.03
	2:1	74.81	68.46
	3:1	74.53	70.26
	4:1	75.16	76.92
	5:1	75.85	70.00
Spectral feature parameters (*r*>0.6)	1:1	74.62	68.46
	2:1	74.04	70.38
	3:1	72.48	66.15
	4:1	74.52	75.00
	5:1	75.08	66.92
Spectral feature parameters (*r*>0.7)	1:1	70.26	67.95
	2:1	71.15	67.31
	3:1	70.26	66.15
	4:1	70.19	68.59
	5:1	70.92	68.46
Spectral feature parameters (*r*>0.8)	1:1	38.97	38.72
	2:1	38.08	38.46
	3:1	38.97	38.46
	4:1	36.70	41.03
	5:1	39.08	37.69

In the case of the disease identification DPLS model built based on the original spectral reflectance data, the original spectral reflectance in the visible region, the original spectral reflectance in the near infrared region, the first derivatives of the original spectral reflectance, the second derivatives of the original spectral reflectance or the logarithms of the reciprocals of the original spectral reflectance, the identification accuracy for the training set was more than 99%. Except that the identification accuracies of the DPLS models built based on the original spectral reflectance in the near infrared region with the ratios of 1:1, 2:1, 3:1 and 5:1 for the testing sets were lower than 98% (the accuracies were 95.90%, 97.31%, 96.92% and 97.69%, respectively), the identification accuracies of the other DPLS models for the testing sets were more than 98%. Especially, for the DPLS models built based on the logarithms of the reciprocals of the original spectral reflectance with the ratios of 4:1 and 5:1, the identification accuracies for the training sets and the testing sets were all 100.00%.

In the case of the disease identification SVM models built based on the original spectral reflectance data, the original spectral reflectance in the visible region, the original spectral reflectance in the near infrared region, the first derivatives of the original spectral reflectance, the second derivatives of the original spectral reflectance and the logarithms of the reciprocals of the original spectral reflectance, respectively, the identification accuracies for the training sets were more than 99%. Except that the identification accuracies of the SVM models built based on the original spectral reflectance in the near infrared region with the ratios of 1:1, 2:1, 3:1 and 4:1 for the testing sets were lower than 95% (the accuracies were 93.33%, 93.46%, 93.33% and 92.31%, respectively), the identification accuracies of the other SVM models for the testing sets were more than 95%. Especially, for the SVM model built based on the first derivatives of the original spectral reflectance with the ratio of 5:1 and the SVM models built based on the logarithms of the reciprocals of the original spectral reflectance with the ratios of 2:1, 4:1 and 5:1, the identification accuracies for the training sets and the testing sets were all 100.00%. The identification accuracies of the models built based on the selected spectral feature parameters were very low, and the identification accuracies of the DPLS model and the SVM model built based on NDVI (*r*>0.8) for the training sets and the testing sets were the lowest.

### Severity Inversion of Wheat Stripe Rust and Leaf Rust Based on the Hyperspectral Data at the Leaf Level

#### Severity Inversion of Wheat Stripe Rust and Leaf Rust by Modeling Based on LOOCV

When LOOCV was used as the validation method, the results of the severity inversion models of wheat stripe rust and wheat leaf rust built based on the original spectral reflectance data, the original spectral reflectance in the visible region, the original spectral reflectance in the near infrared region, the first derivatives of the original spectral reflectance, the second derivatives of the original spectral reflectance, the logarithms of the reciprocals of the original spectral reflectance and the selected spectral feature parameters, respectively, were shown in Table 6. As shown in Table 6, the *R*^2^ values were more than 0.90 and the RMSE values were lower than 0.16 for the disease severity inversion QPLS models of wheat stripe rust except the QPLS models built based on the first derivatives of the original spectral reflectance and NDVI (*r*>0.8). The *R*^2^ values were more than 0.82 and the RMSE values were lower than 0.17 for the disease severity inversion SVR models of wheat stripe rust except the SVR model built based on the selected spectral feature parameters as *r*>0.6. For the disease severity inversion QPLS models of wheat leaf rust, all the values of *R*^2^ were more than 0.85. Especially, the optimal *R*^2^ values were obtained when the QPLS models of wheat leaf rust were built based on the first derivatives of the original spectral reflectance and the second derivatives of the original spectral reflectance, respectively, and they were 0.9788 and 0.9814, respectively. However, the values of the corresponding RMSE of 0.3567 and 0.4191 were the highest. The values of *R*^2^ were more than 0.83 and the values of RMSE were lower than 0.15 for the disease severity inversion SVR models of wheat leaf rust except the SVR model built based on NDVI (*r*>0.8). Especially, the values of *R*^2^ were more than 0.90 and the values of RMSE were lower than 0.13 for the disease severity inversion SVR models of wheat leaf rust built based on the selected spectral feature parameters as *r*>0.5 and *r*>0.7, respectively. The values of *R*^2^ and RMSE were 0.9429 and 0.1396 for the optimal model for disease severity inversion of wheat stripe rust using QPLS, and those were 0.9402 and 0.0921 for the optimal model for disease severity inversion of wheat stripe rust using SVR. In the case of wheat leaf rust, the values of *R*^2^ and RMSE were 0.9304 and 0.1462 for the optimal model for disease severity inversion using QPLS, and those were 0.9084 and 0.1098 for the optimal model for disease severity inversion using SVR.

**Table 6:** The results of the disease severity inversion models of wheat stripe rust and leaf rust by LOOCV.

Modeling data	Disease category	QPLS model		SVR model	
		*R*^2^	RMSE	*R*^2^	RMSE
Original spectral reflectance data	Wheat stripe rust	0.9138	0.1149	0.9204	0.1017
	Wheat leaf rust	0.8950	0.1346	0.8863	0.1298
Original spectral reflectance in the visible region	Wheat stripe rust	0.9201	0.0982	0.8362	0.1405
	Wheat leaf rust	0.9055	0.1067	0.8809	0.1198
Original spectral reflectance in the near infrared region	Wheat stripe rust	0.9001	0.1097	0.9036	0.1078
	Wheat leaf rust	0.8600	0.1299	0.8380	0.1397
First derivatives of the original spectral reflectance	Wheat stripe rust	0.8964	0.1340	0.9401	0.0953
	Wheat leaf rust	0.9788	0.3567	0.8457	0.1358
Second derivatives of the original spectral reflectance	Wheat stripe rust	0.9107	0.1507	0.9327	0.0974
	Wheat leaf rust	0.9814	0.4191	0.8364	0.1421
The logarithms of the reciprocals of the original spectral reflectance	Wheat stripe rust	0.9197	0.1147	0.9402	0.0921
	Wheat leaf rust	0.9304	0.1462	0.8583	0.1151
Spectral feature parameters (*r*>0.5)	Wheat stripe rust	0.9306	0.1303	0.8215	0.1530
	Wheat leaf rust	0.8913	0.1322	0.9084	0.1098
Spectral feature parameters (*r*>0.6)	Wheat stripe rust	0.9251	0.1323	0.7647	0.1545
	Wheat leaf rust	0.8775	0.1306	0.8820	0.1132
Spectral feature parameters (*r*>0.7)	Wheat stripe rust	0.9429	0.1396	0.8901	0.1590
	Wheat leaf rust	0.8775	0.1306	0.9023	0.1211
Spectral feature parameters (*r*>0.8)	Wheat stripe rust	0.8672	0.1539	0.9250	0.1647
	Wheat leaf rust	0.8580	0.1366	0.7203	0.2001

#### Severity Inversion of Wheat Stripe Rust and Leaf Rust by Modeling Based on External Validation

When external validation method was used for modeling, the effects of different ratios between training sets and testing sets on the severity inversion QPLS models of wheat stripe rust were shown in Table 7, and the effects of different ratios between training sets and testing sets on the severity inversion QPLS models of wheat leaf rust were shown in Table 8. As shown in Tables 7 and 8, for both wheat stripe rust and wheat leaf rust, the disease severity inversion QPLS models built based on the original spectral reflectance data, the original spectral reflectance in the visible region, the original spectral reflectance in the near infrared region, the first derivatives of the original spectral reflectance, the second derivatives of the original spectral reflectance and the logarithms of the reciprocals of the original spectral reflectance, respectively, were better than that built based on the selected spectral feature parameters. In addition, the modeling ratios had relatively slight effects on the values of *R*^2^ and RMSE for the training sets and had relatively great effects on the values of *R*^2^ and RMSE for the testing sets. For wheat stripe rust, the disease severity inversion model built based on the original spectral reflectance in the visible region with the ratio of the training set to the testing set equal to 3:1 was the optimal model with an *R*^2^ of 0.9514 and a RMSE of 0.0762 for the training set and an *R*^2^ of 0.9274 and a RMSE of 0.0947 for the testing set. For wheat leaf rust, the disease severity inversion model built based on the original spectral reflectance in the visible region with the ratio of the training set to the testing set equal to 1:1 was optimal. The values of *R*^2^ and RMSE for the training set of this model were 0.9419 and 0.0832, respectively, and those for the testing set were 0.9137 and 0.1026, respectively.

**Table 7:** The effects of different ratios between training sets and testing sets on the disease severity inversion QPLS models of wheat stripe rust.

Modeling data	The ratio of training set to testing set	Training set		Testing set	
		*R*^2^	RMSE	*R*^2^	RMSE
Original spectral reflectance data	1:1	0.9030	0.1075	0.8882	0.1167
	2:1	0.9132	0.1023	0.8975	0.1111
	3:1	0.9061	0.1059	0.9087	0.1062
	4:1	0.9048	0.1071	0.8912	0.1145
	5:1	0.9108	0.1033	0.8971	0.1132
Original spectral reflectance in the visible region	1:1	0.9512	0.0763	0.9035	0.1085
	2:1	0.9185	0.0991	0.9064	0.1062
	3:1	0.9514	0.0762	0.9274	0.0947
	4:1	0.9016	0.1089	0.7798	0.1629
	5:1	0.9445	0.0815	0.9292	0.0939
Original spectral reflectance in the near infrared region	1:1	0.9161	0.1000	0.9132	0.1029
	2:1	0.9223	0.0968	0.9078	0.1054
	3:1	0.9189	0.0985	0.9281	0.0942
	4:1	0.9215	0.0973	0.8938	0.1132
	5:1	0.9196	0.0981	0.9201	0.0997
First derivatives of the original spectral reflectance	1:1	0.9064	0.1056	0.8150	0.1502
	2:1	0.9100	0.1041	0.9024	0.1085
	3:1	0.9205	0.0975	0.8535	0.1345
	4:1	0.9287	0.0927	0.8132	0.1500
	5:1	0.9140	0.1015	0.9131	0.1040
Second derivatives of the original spectral reflectance	1:1	0.9329	0.0894	0.7390	0.1784
	2:1	0.9118	0.1031	0.8758	0.1224
	3:1	0.9124	0.1023	0.8129	0.1520
	4:1	0.9052	0.1069	0.7701	0.1665
	5:1	0.9115	0.1029	0.8674	0.1285
The logarithms of the reciprocals of the original spectral reflectance	1:1	0.9018	0.1082	0.9029	0.1088
	2:1	0.9113	0.1034	0.8696	0.1254
	3:1	0.9063	0.1058	0.8783	0.1226
	4:1	0.9086	0.1050	0.8627	0.1286
	5:1	0.9332	0.0894	0.9412	0.0855
Spectral feature parameters (*r*>0.5)	1:1	0.8420	0.1372	0.7578	0.1718
	2:1	0.8343	0.1413	0.7779	0.1636
	3:1	0.8509	0.1335	0.6815	0.1983
	4:1	0.8413	0.1383	0.7243	0.1823
	5:1	0.8273	0.1438	0.7856	0.1634
Spectral feature parameters (*r*>0.6)	1:1	0.8411	0.1376	0.7564	0.1723
	2:1	0.8128	0.1502	0.7989	0.1557
	3:1	0.8504	0.1337	0.6616	0.2044
	4:1	0.8266	0.1446	0.7330	0.1794
	5:1	0.8096	0.1510	0.8119	0.1530
Spectral feature parameters (*r*>0.7)	1:1	0.8401	0.1380	0.7668	0.1686
	2:1	0.8069	0.1526	0.8073	0.1524
	3:1	0.8501	0.1338	0.6703	0.2018
	4:1	0.8167	0.1487	0.7689	0.1669
	5:1	0.8041	0.1531	0.8280	0.1463
Spectral feature parameter (*r*>0.8)	1:1	0.8061	0.1520	0.7547	0.1729
	2:1	0.7822	0.1620	0.7746	0.1648
	3:1	0.8206	0.1464	0.6601	0.2049
	4:1	0.7798	0.1629	0.7810	0.1625
	5:1	0.7769	0.1634	0.7926	0.1607

**Table 8:** The effects of different ratios between training sets and testing sets on the disease severity inversion QPLS models of wheat leaf rust.

Modeling data	The ratio of training set to testing set	Training set		Testing set	
		*R*^2^	RMSE	*R*^2^	RMSE
Original spectral reflectance	1:1	0.9597	0.0693	0.8613	0.1300
	2:1	0.9234	0.0961	0.8809	0.1198
	3:1	0.9291	0.0920	0.8515	0.1354
	4:1	0.9247	0.0953	0.9021	0.1086
	5:1	0.9295	0.0919	0.8408	0.1408
Original spectral reflectance in the visible region	1:1	0.9419	0.0832	0.9137	0.1026
	2:1	0.9236	0.0960	0.9081	0.1052
	3:1	0.9332	0.0894	0.8875	0.1179
	4:1	0.9176	0.0997	0.9104	0.1039
	5:1	0.9283	0.0927	0.9112	0.1052
Original spectral reflectance in the near infrared region	1:1	0.9346	0.0883	0.8349	0.1419
	2:1	0.9172	0.0999	0.8776	0.1215
	3:1	0.9107	0.1033	0.8390	0.1410
	4:1	0.9045	0.1073	0.8912	0.1145
	5:1	0.9102	0.1037	0.8327	0.1443
First derivatives of the original spectral reflectance	1:1	0.9266	0.0935	0.8824	0.1910
	2:1	0.9197	0.0984	0.8726	0.1727
	3:1	0.9099	0.1038	0.8634	0.1847
	4:1	0.9075	0.1056	0.8321	0.1600
	5:1	0.9082	0.1048	0.8557	0.1897
Second derivatives of the original spectral reflectance	1:1	0.9170	0.0995	0.8432	0.1732
	2:1	0.9037	0.1078	0.8764	0.1347
	3:1	0.9073	0.1052	0.8136	0.1644
	4:1	0.9022	0.1086	0.8469	0.1772
	5:1	0.9014	0.1087	0.8954	0.1421
The logarithms of the reciprocals of the original spectral reflectance	1:1	0.9212	0.0969	0.8354	0.1416
	2:1	0.9140	0.1018	0.8149	0.1494
	3:1	0.9021	0.1082	0.8483	0.1368
	4:1	0.9133	0.1023	0.7878	0.1599
	5:1	0.9098	0.1039	0.7915	0.1611
Spectral feature parameters (*r*>0.5)	1:1	0.8666	0.1261	0.8636	0.1289
	2:1	0.8805	0.1200	0.8313	0.1426
	3:1	0.8692	0.1250	0.8792	0.1221
	4:1	0.8743	0.1231	0.8649	0.1276
	5:1	0.8820	0.1189	0.8106	0.1536
Spectral feature parameters (*r*>0.6)	1:1	0.8617	0.1284	0.8598	0.1307
	2:1	0.8661	0.1271	0.8747	0.1229
	3:1	0.8659	0.1266	0.8730	0.1253
	4:1	0.8658	0.1272	0.8836	0.1184
	5:1	0.8723	0.1237	0.8566	0.1336
Spectral feature parameters (*r*>0.7)	1:1	0.8261	0.1439	0.8742	0.1238
	2:1	0.8489	0.1349	0.8620	0.1290
	3:1	0.8405	0.1381	0.8928	0.1150
	4:1	0.8493	0.1348	0.8717	0.1244
	5:1	0.8562	0.1312	0.8424	0.1401
Spectral feature parameter (*r*>0.8)	1:1	0.6428	0.2063	0.6511	0.2062
	2:1	0.6186	0.2144	0.7006	0.1900
	3:1	0.6443	0.2062	0.6487	0.2083
	4:1	0.6682	0.2000	0.5607	0.2301
	5:1	0.6272	0.2113	0.7418	0.1793

As shown in Tables 9 and 10, for both wheat stripe rust and wheat leaf rust, the disease severity inversion SVR models built based on the original spectral reflectance data, the original spectral reflectance in the visible region, the original spectral reflectance in the near infrared region, the first derivatives of the original spectral reflectance, the second derivatives of the original spectral reflectance and the logarithms of the reciprocals of the original spectral reflectance, respectively, were better than that built based on the selected spectral feature parameters when external validation method was used for modeling. Among the disease severity inversion SVR models of wheat stripe rust, the model built based on the first derivatives of the original spectral reflectance was the best as the ratio of the training set to the testing set was 2:1. And the values of *R*^2^ and RMSE for the training set of this model were 0.9992 and 0.0097, respectively, and those for the corresponding testing set were 0.9456 and 0.0810, respectively. Among the disease severity inversion SVR models of wheat leaf rust, the model built based on the original spectral reflectance in the visible region with the ratio of the training set to the testing set equal to 2:1 was the best with an *R*^2^ of 0.9841 and a RMSE of 0.0438 for the training set and an *R*^2^ of 0.9403 and a RMSE of 0.0848 for the testing set.

**Table 9:** The effects of different ratios between training sets and testing sets on the disease severity inversion SVR models of wheat stripe rust.

Modeling data	The ratio of training set to testing set	Training set		Testing set	
		*R*^2^	RMSE	*R*^2^	RMSE
Original spectral reflectance data	1:1	0.9982	0.0146	0.9009	0.1099
	2:1	0.9974	0.0178	0.8710	0.1247
	3:1	0.9969	0.0193	0.8515	0.1354
	4:1	0.9980	0.0154	0.8488	0.1350
	5:1	0.9877	0.0384	0.8747	0.1249
Original spectral reflectance in the visible region	1:1	0.9257	0.0941	0.8448	0.1376
	2:1	0.9469	0.0800	0.9118	0.1031
	3:1	0.9881	0.0377	0.9073	0.1070
	4:1	0.9895	0.0355	0.8882	0.1161
	5:1	0.9856	0.0415	0.9540	0.0757
Original spectral reflectance in the near infrared region	1:1	0.9852	0.0420	0.9127	0.1032
	2:1	0.9922	0.0306	0.8627	0.1286
	3:1	0.9950	0.0243	0.8556	0.1335
	4:1	0.9961	0.0217	0.8316	0.1425
	5:1	0.9834	0.0446	0.8574	0.1332
First derivatives of the original spectral reflectance	1:1	0.9992	0.0099	0.8938	0.1138
	2:1	0.9992	0.0097	0.9456	0.0810
	3:1	0.9991	0.0103	0.9263	0.0954
	4:1	0.9992	0.0098	0.8791	0.1207
	5:1	0.9992	0.0099	0.9422	0.0849
Second derivatives of the original spectral reflectance	1:1	0.9992	0.0098	0.8731	0.1244
	2:1	0.9988	0.0121	0.9298	0.0920
	3:1	0.9992	0.0100	0.9111	0.1047
	4:1	0.9984	0.0137	0.8402	0.1388
	5:1	0.9989	0.0117	0.9247	0.0968
The logarithms of the reciprocals of the original spectral reflectance	1:1	0.9949	0.0246	0.9177	0.1001
	2:1	0.9642	0.0657	0.9104	0.1039
	3:1	0.9923	0.0304	0.8770	0.1233
	4:1	0.9885	0.0372	0.9347	0.0887
	5:1	0.9912	0.0325	0.9534	0.0762
Spectral feature parameters (*r*>0.5)	1:1	0.8696	0.1246	0.8157	0.1499
	2:1	0.9097	0.1043	0.8056	0.1531
	3:1	0.8690	0.1251	0.7525	0.1748
	4:1	0.8458	0.1363	0.8086	0.1519
	5:1	0.8588	0.1300	0.8308	0.1451
Spectral feature parameters (*r*>0.6)	1:1	0.8737	0.1226	0.7725	0.1665
	2:1	0.8130	0.1501	0.7936	0.1577
	3:1	0.8701	0.1246	0.6823	0.1981
	4:1	0.8291	0.1435	0.7987	0.1558
	5:1	0.8071	0.1520	0.8224	0.1487
Spectral feature parameters (*r*>0.7)	1:1	0.8602	0.1290	0.7828	0.1627
	2:1	0.8239	0.1457	0.8028	0.1542
	3:1	0.8525	0.1328	0.6953	0.1940
	4:1	0.8147	0.1495	0.8101	0.1513
	5:1	0.8057	0.1525	0.8183	0.1504
Spectral feature parameter (*r*>**0**.8)	1:1	0.8604	0.1290	0.7612	0.1706
	2:1	0.8102	0.1513	0.8028	0.1542
	3:1	0.8616	0.1286	0.6573	0.2057
	4:1	0.8048	0.1534	0.8189	0.1477
	5:1	0.8050	0.1528	0.8207	0.1494

**Table 10:** The effects of different ratios between training sets and testing sets on the disease severity inversion SVR models of wheat leaf rust.

Modeling data	The ratio of training set to testing set	Training set		Testing set	
		*R*^2^	RMSE	*R*^2^	RMSE
Original spectral reflectance data	1:1	0.9935	0.0277	0.8511	0.1348
	2:1	0.9436	0.0825	0.9050	0.1070
	3:1	0.9829	0.0452	0.8350	0.1427
	4:1	0.9664	0.0636	0.8824	0.1191
	5:1	0.9685	0.0614	0.8627	0.1307
Original spectral reflectance in the visible region	1:1	0.9465	0.0798	0.8803	0.1208
	2:1	0.9841	0.0438	0.9403	0.0848
	3:1	0.9778	0.0515	0.9200	0.0994
	4:1	0.9729	0.0571	0.9403	0.0848
	5:1	0.9772	0.0522	0.9243	0.0971
Original spectral reflectance in the near infrared region	1:1	0.9830	0.0450	0.8379	0.1406
	2:1	0.9506	0.0771	0.8965	0.1117
	3:1	0.9774	0.0520	0.7997	0.1573
	4:1	0.9563	0.0726	0.8727	0.1239
	5:1	0.9595	0.0696	0.8444	0.1392
First derivatives of the original spectral reflectance	1:1	0.7595	0.1693	0.2260	0.3072
	2:1	0.4017	0.2686	0.3196	0.2864
	3:1	0.8179	0.1475	0.2677	0.3007
	4:1	0.6646	0.2011	0.1697	0.3164
	5:1	0.6004	0.2187	0.4368	0.2648
Second derivatives of the original spectral reflectance	1:1	0.5107	0.2414	0.2154	0.3093
	2:1	0.3255	0.2852	0.2450	0.3017
	3:1	0.4278	0.2615	0.3612	0.2809
	4:1	0.6236	0.2130	0.0625	0.3362
	5:1	0.4027	0.2674	0.3288	0.2891
The logarithms of the reciprocals of the original spectral reflectance	1:1	0.9323	0.0898	0.8929	0.1143
	2:1	0.9510	0.0768	0.9141	0.1017
	3:1	0.9393	0.0851	0.8998	0.1112
	4:1	0.9594	0.0699	0.9090	0.1048
	5:1	0.9343	0.0887	0.8304	0.1453
Spectral feature parameters (*r*>0.5)	1:1	0.9140	0.1013	0.9106	0.1044
	2:1	0.9138	0.1019	0.9264	0.0942
	3:1	0.9197	0.0979	0.8832	0.1201
	4:1	0.9542	0.0743	0.7963	0.1567
	5:1	0.9261	0.0941	0.9014	0.1108
Spectral feature parameters (*r*>0.6)	1:1	0.8980	0.1102	0.9210	0.0981
	2:1	0.9015	0.1090	0.9354	0.0882
	3:1	0.9096	0.1039	0.8828	0.1203
	4:1	0.9281	0.0931	0.7908	0.1588
	5:1	0.9140	0.1015	0.8793	0.1226
Spectral feature parameters (*r*>0.7)	1:1	0.8654	0.1266	0.9367	0.0878
	2:1	0.8826	0.1190	0.9294	0.0922
	3:1	0.8884	0.1155	0.9123	0.1041
	4:1	0.9156	0.1009	0.8128	0.1502
	5:1	0.9034	0.1076	0.9011	0.1110
Spectral feature parameter (*r*>0.8)	1:1	0.6722	0.1976	0.6509	0.2063
	2:1	0.6414	0.2079	0.7414	0.1766
	3:1	0.6806	0.1954	0.6409	0.2106
	4:1	0.7097	0.1871	0.5679	0.2282
	5:1	0.6624	0.2010	0.7844	0.1638

## Discussion

As shown in Fig 1, for both wheat stripe rust and wheat leaf rust, there were differences between the spectral reflectance of the healthy wheat leaves and that of wheat leaves in the incubation period, and there were also differences between the spectral reflectance of the healthy wheat leaves and that of wheat leaves in the diseased period. In addition, there were differences between the spectral reflectance of wheat leaves in the incubation period of stripe rust and that of wheat leaves in the incubation period of leaf rust, and there also were differences between the spectral reflectance of wheat leaves in the diseased period of stripe rust and that of wheat leaves in the diseased period of leaf rust. The differences may be related to the changes of chlorophyll content and cellular structure in the leaves [14, 46]. Plant leaves have a low reflectance in the visible region and a high reflectance in the near infrared region [14, 46, 47]. The low reflectance in the visible region is mainly influenced by pigments in the leaves and the high reflectance in the near infrared region is mainly caused by leaf cellular structure [14, 46, 48, 49]. It was reported that the reflectance in the visible region was negatively related with the chlorophyll content of wheat leaves [50]. The chlorophyll content of wheat leaves in the incubation period gradually increases with the growth of wheat, so the reflectance decreases compared with the reflectance of the healthy wheat leaves [50]. And in the diseased period, a large number of uredinia break through the epidermis of the leaves, and this induces the destruction of the leaf structure and the decrease of the chlorophyll content, resulting in the increase of the spectral reflectance [50]. The specific mechanisms of the reflectance differences are still needed to be further studied.

Plant canopy spectra are influenced by many factors such as geometrical structure and soil background while the hyperspectral measurements are performed under in-field natural conditions [13, 15]. Compared with the canopy spectra, the spectra at the leaf level are more accurate and more reliable. However, it is difficult to accurately acquire spectral data at the leaf level. Usually, the leaves are brought back to a laboratory for spectral measurements. But in this process, water content and cell structure of the leaves will inevitably be affected, thus the accuracy of spectral data will also be influenced. Moreover, indoor spectral measurements are usually performed with the aid of an artificial light source and often require experimental equipments and devices such as integrating sphere and leaf clip. To solve these problems, a novel spectral measuring method named as black-paper-based measuring method was developed in this study. Under the in-field natural conditions, after the collected fresh leaves are pasted side by side on a piece of black paper, the spectral data at the leaf level can be measured by using a portable spectrometer. Using the models built based on the hyperspectral data acquired by using the proposed measuring method, satisfactory results were achieved for both disease identification and disease severity inversion. The results indicated that the so-called black-paper-based measuring method is a low-cost, easily operated and acceptable method. It is simple and convenient. Using this method, the influence of vegetation coverage and geometry structure of plant canopy on spectral data can be avoided. Meanwhile, the reduction of water content and the damage on cell structure in the preservation process of the leaves can also be avoided because the spectral measurements are performed immediately after the leaves are collected. Certainly, this method is only suitable for the spectral measurements at the leaf level. And it is more suitable when the measurements can not be performed without the aid of special equipments and devices such as integrating sphere or leaf clip under outdoor conditions and the accuracy of the acquired spectral data is to some extent required. However, it is obviously not suitable for the spectral measurements in large scale.

Spectral measurements at the leaf scale are usually performed with the aid of special instruments and equipments such as integrating sphere or leaf clip, and sometimes, spectral measurements are performed under indoor illumination while the leaf or the leaves are placed on a black background. It was reported that reflectance measurements of plant leaves were made for extraction of leaf biochemistry information while a leaf disk punched from each leaf was placed into a sample holder with a black background [51]. In a study conducted by Liu et al. [52], indoor measurements of spectra of rice leaves placed in a black observation platform with an approximately zero reflectivity were made under a light source for discriminating rice leaves infected by *Aphelenchoides besseyi*. It was reported that the reflectance spectra at the leaf level for assessment of water content of wheat leaves were measured directly by a spectrometer under a light source in the indoor environment [53]. Moreover, for the leaves with large width of some plants such as cucumber and cotton, the reflectance spectra of individual leaves could be measured directly by a spectrometer with natural illumination [54, 55]. The developed spectral measuring method in this study was implemented based on the use of a piece of black paper as the background under the in-field natural conditions.

The results demonstrated that, except the disease severity inversion models of wheat stripe rust and leaf rust by LOOCV, the models built based on the original spectral reflectance data, the original spectral reflectance in the visible region, the original spectral reflectance in the near infrared region, the first derivatives of the original spectral reflectance, the second derivatives of the original spectral reflectance or the logarithms of the reciprocals of the original spectral reflectance were better than that built based on the selected spectral feature parameters. This may be due to the original spectral reflectance data and the data derived from various transformations including more spectral bands, and thus they contained more information of the healthy wheat leaves and the wheat leaves infected with *Pst* or *Prt* in comparison with the selected spectral feature parameters. It has been proved that modeling accuracy increased with the increase of the number of wavelengths [56].

Although the accuracy is high while the spectral measurements are performed at the leaf level, it is very difficult to apply this spectral measuring method to monitoring plant diseases in large area. Therefore, it is important to find a method that is applicable to monitoring plant diseases in large area with an acceptable accuracy in further studies.

## Conclusions

Wheat stripe rust and leaf rust with the similar symptoms are easily confused by symptom observation. It is of great significance to implement the rapid, accurate and timely identification and assessment of these two diseases for sustainable management of the diseases. A novel hyperspectral data measuring method named as black-paper-based measuring method was developed in this study. Using this method, the hyperspectral data of wheat at the leaf level can be acquired under the in-field natural conditions. The potential impacts produced in the process of taking the leaves back to the laboratory for spectral measurements can be avoid. This method is low-cost and easily operated, and it is an applicable method for spectral measurements at the leaf level. Based on the hyperspectral data acquired by using the black-paper-based measuring method, the built identification models of wheat stripe rust and leaf rust could identify the healthy wheat leaves, wheat leaves in incubation period of stripe rust, wheat leaves in diseased period of stripe rust, wheat leaves in incubation period of leaf rust and wheat leaves in diseased period of leaf rust with high identification accuracies, and the satisfactory inversion results were obtained using the built disease severity inversion models. When LOOCV was used as the validation method, the accuracies of the optimal DPLS model and the optimal SVM model for discriminating the two diseases were more than 99%; for wheat stripe rust, the values of *R*^2^ of the optimal disease severity inversion QPLS model and the optimal disease severity inversion SVR model were more than 0.94, and the RMSE values were less than 0.15; for wheat leaf rust, the *R*^2^ values of the optimal disease severity inversion QPLS model and the optimal disease severity inversion SVR model were more than 0.90, and the RMSE values were less than 0.15. When external validation was used for modeling, the accuracies of the optimal disease identification models for the training sets and the testing sets were all 100.00%; for both wheat stripe rust and leaf rust, the values of *R*^2^ of the optimal disease severity inversion QPLS model and the optimal disease severity inversion SVR model for the training sets were more than 0.94 and the corresponding RMSE values were less than 0.10, and the values of *R*^2^ for the testing sets were more than 0.90 and the corresponding RMSE values were less than 0.15. It was indicated that identification and assessment of wheat stripe rust and wheat leaf rust based on the hyperspectral data acquired by using the developed black-paper-based measuring method are feasible. In this study, a scientific basis was provided for implementing satellite remote sensing monitoring of these two kinds of wheat diseases.

## Supporting Information

S1 Data The spectral reflectance of five categories of wheat leaves after averaging the original spectral data according to their categories. (XLSX)
